# A double-blind, randomized, multicentric, placebo-controlled clinical trial of antarth, a phytomedicine, in the treatment of osteoarthritis

**DOI:** 10.4103/0253-7613.75674

**Published:** 2011-02

**Authors:** Anil K. Gupta, Kirankumar Acharya, Parag S. Sancheti, Ramchandra S. Joshi

**Affiliations:** Department of Orthopaedics, G.S.V.M. Medical College, Kanpur – 208 002, Uttar Pradesh, India; 1 Department of Orthopaedics, K.G. Medical College and Hospital, Manipal – 576 104, Karnataka, India; 2 Sancheti Institute for Orthopaedics and Rehabilitation, 16, Shivaji Nagar, Pune – 411 005, Maharashtra, India; 3 14 Shiv Shanti, Juhu Versova Link Road, Andheri (west), Mumbai - 400 053, Maharashtra, India

**Keywords:** Functional disability, global assessment, rescue medication, Visual Analog Scale

## Abstract

**Objective:**

To test Antarth, a polyherbal phytomedicine, for its efficacy and safety in patients with osteoarthritis (OA) and compared with placebo.

**Material and Methods:**

A total of 90 male or female adult patients who were diagnosed clinically and radiologically with OA were recruited in the study. Antarth or placebo was given 2 capsules b.i.d. for 3 months and the patients were assessed every month for its efficacy. Diclofenac sodium was allowed to be taken as rescue medication.

**Results:**

After 3 months of treatment, the reduction in severity of pain on Visual Analog Scale (VAS) was more in Antarth group compared to placebo but the difference between the two groups was not significant. However, pain during functioning of disabled joints while walking distance, squatting, sitting cross-legged and climbing steps were significantly reduced in Antarth group compared to placebo (*P* < 0.05). Reduction in consumption of rescue medication, diclofenac sodium, was more in Antarth than in placebo group.

**Conclusions:**

In Patients’ Global Assessment, patients treated with Antarth were more satisfied than the ones treated with placebo. Observations were similar in Physicians’ Global Assessment too. There were no adverse events in both the groups.

## Introduction

Due to changed lifestyle such as lack of exercise and changed food habits, lifestyle disorders such as arthritis, asthma, hypertension and diabetes are very common, particularly amongst urban population. Osteoarthritis (OA) is a progressive rheumatic disease characterized by the degeneration of articular cartilage. It is the most common of all rheumatic disorders.[1] Drug therapy includes non-opiod analgesics such as paracetamol, non-steroidal anti-inflammatory drugs (NSAIDs), topical analgesics, opioid analgesics and intra-articular steroid injection. Such treatments may prove ineffective in some patients and NSAIDs often have serious adverse effects.[2,3] Gastrointestinal (GI) complications are frequently reported with NSAIDs, with 12,000 hospitalizations and about 2000 deaths attributed to NSAID use in the United Kingdom every year.[2–5] Hence, there appears to be a need for drugs with good efficacy and low toxicity in the treatment of OA. Specifically, there is a need for safe and effective medication for patients who do not respond well to the conventional medical therapy. Such patients are turning increasingly to complimentary/alternative medicine.

Antarth is one such polyherbal Ayurvedic phytomedicine used for the treatment of arthritis. The present study was undertaken to evaluate its efficacy and safety in patients of OA.

## Material and Methods

Male or female patients above the age of 18, diagnosed clinically and radiologically as osteoarthritis and suffering for more than 2 years, were recruited in the study. Predefined clinical features were pain in the knees, swelling, stiffness and joint tenderness in persons of age more than 45 years, complaining of difficulty in walking for more than 2 years.

Radiological features were any one or more radiological signs like anterior spiking of patella, subchondral sclerosis, lipping or spurring of joint margin, osteophytes and reduction of medial joint space present.[6] Patients suffering from hyperacidity, peptic ulcers, gastric ulcers, gastritis, renal and liver disorders, other types of arthritis, those who are on long-term treatment of steroids or had surgical interventions were excluded. Pregnant and lactating women were also not included. Institutional Ethics Committee approvals were obtained from the all the three centers. One Ayurveda person was constantly associated with this study during the whole period and analysis of study of all the three centers. This Ayurvedic physician was collaborating with all the three centers.

Patients selected as per the criteria mentioned above, were recruited in the study after obtaining written informed consent and allotted to treatment group as per randomization. Routine blood investigations such as complete blood count, liver function test, and renal function test were carried out before starting the treatment. X-ray of the affected knee – AP standing and lateral view – was also done to confirm the diagnosis of OA and to assess cartilage space changes. Antarth or placebo was given in a dose of two capsules b.i.d. for a period of 3 months. Diclofenac sodium 50 mg b.i.d. and Ranitidine 150 mg o.d. was allowed to be taken as rescue medication as and when required and the patients were asked to keep a record of number of tablets taken per month. Patients were asked to report to the study center every month and the compliance of medication was tested by counting the remaining tablets. Efficacy parameters assessed were i) severity of pain measured on Visual Analog Scale (VAS) of 0–10 and it was done in two ways; ii) Functional Disability of joint was assessed by measuring pain while a) walking distance, b) squatting, c) crossing legs and knees, and d) climbing steps and scored as 0 no pain, 1 mild pain, 2 moderate pain, 3 severe pain and 4 acute pain. After 3 months of treatment, again the blood investigations were carried out and Patients’ and Physicians’ Global Assessment was made. At all the three centers, opinion from radiologists was obtained regarding the pre and post treatment X-rays.

## Results

Out of the 90 patients recruited in the study, 2 dropped out due to personal reasons. The results obtained from remaining 88 patients who completed 3 months of treatment were analyzed. The distribution of patients in both the groups was equal and the demographic data with respect to sex, age and body weight were matching [Table 1]. The vitals and the systemic clinical examination were normal in all the patients and were matching in both the groups.

**Table 1:** Demographic data of the patients

*Parameter*	*Antarth (n = 44)*	*Placebo (n = 44)*
Males	17	17
Females	27	27
Age (years) (mean ± SEM)	53.5 ± 1.2	51.9 ± 1.4
Body weight (kg) (mean ± SEM)	67.1 ± 1.5	67.8 ± 1.6

The scores at the basal level for all the parameters of efficacy were similar in both the groups [Table 2]. Severity of pain was significantly reduced after 3 months treatment in Antarth group (*P* < 0.05) but was not significantly reduced in placebo group. There was a reduction of 35.8% in mean score for walking distance in Antarth group as compared to placebo group which showed a reduction of 32.6%. Pain while squatting was 1.9 ± 1.0 before treatment and 1.30 ± 0.8 after treatment, which was a reduction of 30.1% in Antarth group, whereas in placebo group the corresponding values were 2.0 ± 1.1 and 1.7 ± 1.1, respectively, which was a reduction of 15.6%. Similarly, pain scores while crossing legs before and after treatment were 1.8 ± 1.1 and 1.2 0.8, respectively, a reduction of 29.4% for Antarth, and for placebo group they were 1.8 ± 1.1 and 1.5 ± 1.1, respectively, a difference of 16.8%. Scores for pain while climbing steps before and after treatment were 1.5 ± 0.9 and 0.9 ± 0.8, respectively, a reduction of 43.4% for Antarth, and for placebo they were 1.8 ± 0.9 and 1.3 ± 1.1, respectively, a reduction of 27.8%. The difference in scores before and after treatment in Antarth group was significant in all the parameters (*P* < 0.05), whereas in placebo group it was not significant.

**Table 2:** Effect on various symptoms before and after treatment by Antarth and placebo in osteoarthritis of knee

*Parameter*	*Antarth*		*Placebo*	
	*Before treatment*	*After treatment*	*After treatment*	*After treatment*
Severity of pain	5.8 ± 2.3	4.7 ± 2.0*	5.9 ± 2.3	4.8 ± 2.2
Functional Disability of joint				
Pain while walking distance	1.3 ± 0.9	0.9 ± 0.8*	1.0 ± 1.0	0.7 ± 0.8
Pain while squatting	1.9 ± 1.0	1.3 ± 0.8*	2.0 ± 1.1	1.7 ± 1.0
Pain while crossing legs	1.8 ± 1.1	1.2 ± 0.8*	1.8 ± 1.1	1.5 ± 1.1
Pain while climbing steps	1.5 ± 0.9	0.9 ± 0.8*	1.8 ± 0.9	1.3 ± 1.1*
Consumption of NSAIDs tablets/month	22.7 ± 29.5	11.1 ± 18.6*	21.1 ± 28.6	4.73 ± 9.88

Values indicate mean ± SE;

* Significantly different from basal value (*P* < 0.05). There were significant differences in the scores of pain while squatting and while climbing steps (*P* < 0.05) between the two groups, while in other activities, the scores were not statistically significant although the values were higher in placebo group that in Antarth group; Test: Analysis of variance (ANOVA) single factor

Consumption of rescue medication, diclofenac sodium, in Antarth group, before treatment was 32.73 tablets per month, which reduced to 29.52 tablets per month after treatment with Antarth, which was a reduction of 34.37%, whereas in placebo group, it was 21.14 tablets per month before treatment and 4.73 tablets per month after treatment, which was a reduction of 22.38%. Though the difference within the group was significant, it was not significant between the groups.

**Figure 1:**
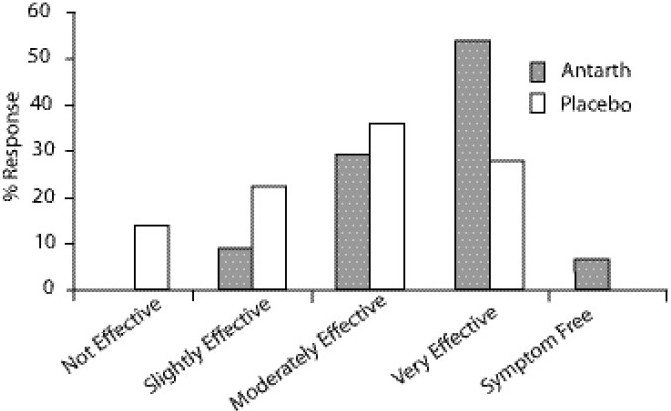
Patient’s Global Assessment.

In Patients’ Global Assessment of efficacy of Antarth, almost all the patients (100%) were satisfied with the treatment with different grades, and none of them said it was not effective. But in placebo group, about 13% of the patients were not satisfied and said it was not effective. The satisfactory levels were as follows. About 6.82% of patients treated with Antarth were symptom free by the end of 3 months treatment, whereas none of the patients in placebo group were symptom free [Figure 1]. About 54.55% of patients in Antarth group said the treatment was very effective, 29.55% said it was slightly effective and none of the patients said it was not effective, whereas the corresponding response in placebo group was very effective (27.27%), moderately effective (36.36%), slightly effective (22.73%) and not effective (13.64%).

**Figure 2:**
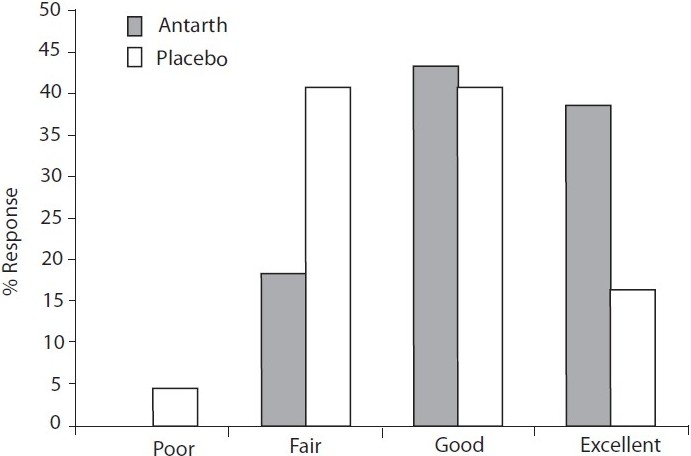
Physician’s Global Assessment.

Similar response was reflected in physicians’ assessment. According to Physicians’ Global Assessment, efficacy of Antarth was as follows: Excellent (38.64%), Good (43.18%), Fair (18.18%) and Poor (0%). Corresponding values for placebo were 15.91, 40.91, 38.64 and 4.55%, respectively [Figure 2].

None of the patients in both the groups reported any clinical adverse event or showed any change in laboratory investigations after treatment.

## Discussion

Although there is no known cure for OA, treatment designed for the individual patient can reduce pain, maintain and/or improve joint mobility and limit functional impairment. The goals of contemporary management of the patient with OA include control of pain and improvement in function and health-related quality of life, with avoidance of toxic effects of therapy. Individuals with OA of the lower extremity may have limitations that impair their ability to perform activities of daily living (ADLs), such as walking, bathing, dressing, use of the toilet, and performing household chores. While physical therapy and occupational therapy play central roles in the management of patients with functional limitations, use of pharmacotherapy is essential to reduce pain during these activities. Most commonly used drug for this is paracetamol. But the drug is known to produce hepatotoxicity at higher doses and hence cannot be used for a longer time and in patients with impaired liver function and in chronic alcoholics.[7–9] Alternatively, NSAID agents are the drugs of choice in the treatment of OA. However, these drugs also have serious adverse effects on GI systems, which are very common. They produce severe acidity leading to ulceration and bleeding. Data from epidemiologic studies show that among persons of age ∅65 years, 20–30% of all hospitalizations and deaths due to peptic ulcer disease were attributable to therapy with NSAIDs.[10–12] More recently, cyclooxygenase-2 (COX-2) inhibitors like celecoxib and rofecoxib have been shown to be more effective and have lesser side effects on GI system than NSAIDs.[13,^14^] However, COX-2-specific inhibitors can cause renal toxicity. Caution must be exercised, therefore, if they are used in patients with hypertension, congestive heart failure, or mild-to-moderate renal insufficiency; they should not be used in patients with severe renal insufficiency.

Arthritis is a chronic disease with no cure but only symptomatic relief got through the use of suitable medications. But the medicines currently available are not safe for long-term use. Hence there is a need for an effective and safe remedy. The alternative systems of medicine claim to have such remedies. Antarth is one such phytomedicine developed on the principles of Ayurveda for the treatment of arthritis.

In OA, the chief complaint of the patients is the pain experienced during performing of some daily routine activities such as walking, sitting cross-legged, climbing steps, etc. These activities are hampered due to pain which affects the quality of life they lead. They are unable to perform their routine activities properly. Reduction of pain while performing these activities improves their lifestyle. The pain during activities like crossing legs, squatting and climbing steps was significantly reduced in Antarth group as compared with placebo group. Pain while crossing legs was reduced by 34.2% in Antarth group and by 16.8% in placebo group after treatment; during squatting it was reduced by 38.1% in Antarth group and 15.6% in placebo group; and while climbing steps it was reduced by 53.5% in Antarth group and 27.8% in placebo group. In all the three activities, the difference in reduction of pain after treatment between the two groups was significant (*P* < 0.05). This indicates that Antarth has a better effect in reducing the pain in patients while performing these activities than placebo. Thus, it helps the patients in performing their day-to-day activities in a better way, thereby improving their quality of life.

The fact that the quality of life is improved is evident from the Patient’s Global Assessment. About 7% of the patients treated with Antarth were totally symptom free by the end of 3 months treatment, whereas none were symptom free in placebo group. More than 55% of the patients treated with Antarth said it was very effective and about 40% said it was mild to moderately effective. Contrary to this, the patients treated with placebo were not fully satisfied with the treatment. About 28% of the patients treated said it was very effective and about 60% said it was mild to moderately effective. This indicates that the patients treated with Antarth were satisfied with the treatment. Similarly, Physician’s Global Assessment about the efficacy and safety of Antarth was better than that of placebo.

Unlike the modern medicines which relieve only the symptoms of the disease, Ayurvedic medicines are claimed to be treating the cause of the disease, thereby relieving the symptoms. Although there is no evidence to this from modern perspectives, it is amply described in ancient Ayurvedic texts. This, however, needs to be confirmed using modern research methodologies.
